# Diagnostic dilemma: Altered sensorium while taking hot water bath

**DOI:** 10.4103/0972-2327.48870

**Published:** 2009

**Authors:** Arun Garg, Anil K. Jain

**Affiliations:** Max Healthcare, B 306, Anandlok Society, Mayur Vihar Phase 1, Delhi - 110 091, India. E-mail: gargarungarg@hotmail.com; 1 Jain Neurocentre, B 306, Anandlok Society, Mayur Vihar Phase 1, Delhi - 110 091, India.

Sir,

It was interesting to read article “Unexplained neurological events during bathing in young people: Possible association with the use of gas geysers”.[1] We had a somewhat similar experience. From November 2005 to January 2008 we have come across 24 patients (age 12 – 45 years) who became confused or comatose while taking hot water bath in winter season. None of them had past history of seizure disorder. The gas geysers were fitted in poorly ventilated bathrooms. In every case family member had to break open the door of bathroom to rescue the patient. Eight patients were comatose when brought to the hospital. Of remaining 16 patients, 10 were in delirious state while six had regained normal sensorium by the time they reached to the hospital. Four patients could vaguely recall feeling of suffocation, dizziness and generalized weakness before loosing consciousness. But none reported any foul smells, or other premonitory symptoms. Their vital parameters were normal except tachycardia up to 120/m and tachypnea up to 30/m was noted. Oxygen saturation was 95-100%. Color of skin was normal in all the cases. There was no odor in their breath. No tongue bite or other injury marks seen. In eight patients, pupils were semi dilated and sluggishly reacting. Fundus examination was normal. There were neither lateralizing signs nor any sign of meningeal irritation present. Dystonic posturing was seen in 5 cases. Hemogram, metabolic profile, MRI-Head and EEG were normal in all the cases. Arterial blood gas (ABG) analysis was done in 18 patients and was found to be normal. All patients were treated with oxygen inhalation. Total duration of unconsciousness or confusion after detection varied from 1 hour to 7-8 hours. All patients made complete recovery.

Besides confusion and convulsions, severe neurological manifestations may occur after days or even weeks after an acute poisoning or after long term repeated exposure. Common problems encountered are difficulty with memory, dementia, Parkinson-like syndromes and cortical blindness.[2] Binding of carbon monoxide (CO) to Hb causes retention of oxygen that would otherwise be delivered to the tissue. Blood oxygen content is actually increased but none is given to the tissues.[3] Because pulse oximeter testing and ABG analysis does not reflect tissue hypoxia, these tests are of little use to screen or diagnose CO poisoning.[4] Levels of carbon monoxide bound in the blood can be determined by measuring carboxyhemoglobin. Serious toxicity is often associated with levels above 25%.[5] Hyperbaric oxygen is also used in the treatment of CO poisoning, which increases carboxyhemoglobin dissociation to a greater extent than normal oxygen.[6]

If no other alternative is available, install geyser outside and give only the hot water outlet pipe in the bathroom. Otherwise one should ensure cross ventilation in the bathroom and install CO detectors. CO can easily be detected by the filtering paper impregnated by the solution of palladium chloride which turns black on exposure to CO.
